# The Cation Distributions of Zn-doped Normal Spinel MgFe_2_O_4_ Ferrite and Its Magnetic Properties

**DOI:** 10.3390/ma15072422

**Published:** 2022-03-25

**Authors:** Xue Zeng, Zhipeng Hou, Jiaqi Ju, Lei Gao, Junwei Zhang, Yong Peng

**Affiliations:** 1 School of Mathematics and Physics, Lanzhou Jiaotong University, Lanzhou 730070, China; zengxue@lzjtu.edu.cn (X.Z.); 1194014153jjq@gmail.com (J.J.); 2 Guangdong Provincial Key Laboratory of Optical Information Material and Technology & Institute for Advanced Materials, South China Academy of Advanced Optoelectronics, South China Normal University, Guangzhou 510006, China; houzp@m.scnu.edu.cn; 3 Electron Microscopy Centre of Lanzhou University and Key Laboratory of Magnetism and Magnetic Materials of the Ministry of Education, School of Materials and Energy, Lanzhou University, Lanzhou 730000, China; lgao19@lzu.edu.cn

**Keywords:** ferrites, nanofibers, C_S_-STEM characterization, atomic structure, magnetic properties

## Abstract

Determining the exact occupation sites of the doping ions in spinel ferrites is vital for tailoring and improving their magnetic properties. In this study, the distribution and occupation sites of cations in MgFe_2_O_4_ and Zn-doped MgFe_2_O_4_ ferrite are imaged by Cs-STEM. The experimental STEM images along [001], [011] and [111] orientations suggest that the divalent Mg^2+^ cations occupy all A sites, and the trivalent Fe^3+^ cations occupy all B sites in MgFe_2_O_4_ ferrite prepared by electrospinning, which is consistent with the normal spinel structure. We further clarify that the preferred sites of dopant Zn^2+^ ions are Fe^3+^ crystallographic sites in the Zn-doped MgFe_2_O_4_ ferrite nanofibers. Magnetic measurements show that Zn doping affects the spin states of the Fe^3+^, and the Fe^3+^-O^2−^-Fe^3+^ super-exchange interaction leads to enhancements in the magnetization and reduction in the Curie temperature. Our work should contribute a significant step toward eventually realizing the practical application of doped spinel ferrites.

## 1. Introduction

The direct imaging of atomic structures, especially in the site preference of substituted ions, is vital for magnetic materials to correctly explain their magnetic performance and provide guidance for potential commercial applications, including microwave devices, magnetic storage media, ferrofluids and biomedical devices [1,2,3,4,5,6,7]. It is known that the structural, electrical and magnetic properties of materials are highly sensitive to the conditions of their preparation, compositions and magnetic interactions, which strongly depend on the distribution of cations [8,9,10,11,12]. Magnetic spinel ferrites are of great interest in addressing the fundamental relationship between magnetic properties and their crystal structures, and they have received significant attention for their unique magnetic properties, such as a high Curie temperature, large magnetocrystalline anisotropy, a large Kerr effect and low magnetic losses [13,14,15,16]. Spinel ferrites are represented as [M_1−*x*_Fe*_x_*]_tet_[M*_x_*Fe_(2−*x*)_]_oct_O_4_ with two different co-ordination polyhedra for cations, namely tetrahedral and octahedral sites, where x represents the degree of inversion if (x = 0) is normal, (x = 1) is inverse and (0 < *x* < 1) is partially inversed. Twenty-four metallic ions occupied two interstitial crystallographic sites, including tetrahedral and octahedral sites. They showed a distribution of parallel and antiparallel magnetic moments in two sub-lattices, which resulted in a net magnetic moment per formula unit and eventually determined the unique magnetic properties of the spinel ferrites. Their magnetic properties could be systematically varied by changing the identity of the divalent M^2+^ cation or by a partial substitution while maintaining the basic crystal structure.

Biocompatible magnesium ferrite (MgFe_2_O_4_), an important functional soft magnetic (*M*_s_ ~ 27.8 emu/g; *H*_c_ ~ 9 Oe; *T*_c_ ~ 713 K) [17,18] and an n-type semiconductor material with a direct band gap of 1.9 eV [19], is an important member of the spinel family. It is widely used in transformer cores, humidity sensors, catalysts, coil cores, heterogeneous catalysis and sensors [20,21]. Recently, it has been reported in the literature that this ferrite could be applied to thermal coagulation treatment using an alternating magnet [22]. Many attempts have been made to reduce their cost and further improve their magnetic performance. Among these, ion doping has proven to be an effective method to tailor the magnetic performance of spinel ferrites [23,24]. The magnetic and dielectric properties of substituted ferrites directly depend on the electronic configuration of the dopant cations and on their preference for occupying the different Fe^3+^ sub-lattices of the spinel structure, such as Zn_x_Mg_1−x_Fe_2_O_4_ [25,26,27,28]. Non-magnetic Zn^2+^ ions are selected as doping ions to further improve the magnetic performance of the MgFe_2_O_4_ ferrite. However, the preferred site of the dopant Zn atoms is indistinct, so a satisfactory explanation of the magnetic properties of Zn-doped MgFe_2_O_4_ is still a matter of debate. Therefore, it is necessary to image their specific distributions in a sub-ångstrom resolution. Among the techniques for detecting the distributions and the preferred site of the doping ions [29,30,31,32,33,34,35], aberration-corrected transmission electron microscopy (Cs-corrected TEM) and scanning transmission electron microscopy (Cs-corrected STEM) equipped with energy-dispersive X-ray analysis (EDX) have recently made significant progress in space and time resolution. These techniques achieve a surprisingly high spatial resolution below 0.5 Å and readily realize a direct imaging and electronic-state detection of individual atoms and even chemical bonds. In this study, we employed Cs-STEM to systematically study the microstructure of MgFe_2_O_4_ nanofibers and the influence of the substitution and occupancy of doped Zn^2+^ on their magnetic properties. 

In this study, the occupation sites and the occupancies of trace dopants in Zn-doped MgFe_2_O_4_ nanofibers were directly and precisely observed by Cs-STEM, which demonstrated that dopant Zn^2+^ cations preferred to randomly occupy Fe^3+^ crystallographic sites rather than Mg^2+^ sub-lattices to modify magnetic properties. The findings indicated that the Zn^2+^ substitution results in an increase in magnetization and a decrease in the Curie temperature. These results offer insights into interactions between dopant atoms and the magnetic properties in spinel structures and provide guidance for designing magnetoelectric multiferroics applications.

## 2. Materials and Methods

Synthesis of MFO and MZFO nanofibers. Single-particle-chain normal spinel MgFe_2_O_4_ (MFO) and MgZn_0.2_Fe_1.8_O_4_ (MZFO) nanofibers were synthesized by electrospinning. A typical precursor solution was used (A.R., Alfa-Aesar Inc., Ward Hill, NJ, USA), which contained 0.1 mmol of magnesium nitrate hexahydrate, 0.2 mmol of iron nitrite nonahydrate (0.1 mmol magnesium nitrate hexahydrate, 0.18 mmol iron nitrite nonahydrate and 0.02 mmol zinc nitrate hexahydrate); 5 mL of N, N-dimethyl formamide (DMF, A.R., Tianjin Chemical Corp., Tianjin, China) was dissolved into a glass container marked as A solution. Next, 1 mL of A solution, 1 mL of ethanol alcohol and 0.1786 g of poly vinylpyrrolidone (PVP, Mw ≈ 1,300,000, Alfa-Aesar Inc., Ward Hill, NJ, USA) were dissolved into a 5 mL vessel combined with sufficient stirring for 4~5 h. A typical precursor solution was successfully obtained. The electrospinning process was performed with 18 kV DC voltage with a 15 cm gap between the needle tip (Jinan Qinlu Pharmaceutical Technology Co., Ltd., Jinan, China) and the collector; the feed rate was 0.4 mL⋅h^−1^. The electrospun polymer composite nanofibers were first heated at 200 °C for 2 h and then calcined at 900 °C for 3 h with a heating rate of 1 °C⋅min^−1^ in air. The samples were finally cooled to room temperature with the same rate of 1 °C⋅min^−1^.

Characterization of MFO and MZFO nanofibers. The morphology and atomic-level crystal structures of the individual single-particle-chain MFO and MZFO nanofibers were systemically characterized with an aberration-corrected scanning transmission electron microscope (FEI Titan Cubed Themis G2 300, FEI, Hillsboro, OR, USA), which operated at 300 kV and was equipped with a monochromator, Gatan image filter (GIF Quantum ER/965, Gatan, CA, USA), EDX (Bruke EDX, Bruke, VA, USA). And an X-ray diffraction instrument (XRD, Philips X’pert ProMPD, Almelo, The Netherlands). Magnetic properties were investigated using a superconducting quantum interference magnetometer (SQUID, MPMS XL-7, Quantum Design, San Diego, CA, USA) and a magnetic property measurement system (MPMS, SQUID-VSM, Quantum Design, San Diego, CA, USA).

## 3. Results and Discussions

**Figure 1:**
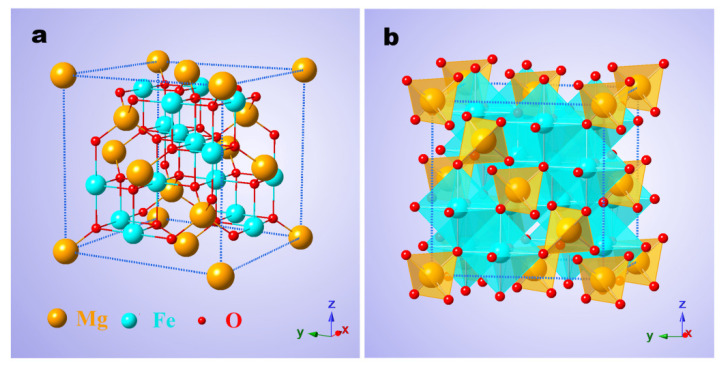
Crystal structure of normal spinel MFO ferrite. (**a**) Ball-and-stick model: the cyan and orange spheres represent Fe cations at B sites and Mg cations at A sites, respectively, and O anions are in red. (**b**) Polyhedral model depicting an FCC cubic network of O anions with Mg^2+^ and Fe^3+^ cations. The tetrahedral A sites and octahedral B sites are marked in orange and cyan, respectively.

Single-particle-chain MgFe_2_O_4_ nanofibers (MFO) and Zn-doped MFO nanofibers (MZFO) were prepared using the electrospinning method. All nanofibers had a continuous structure and uniform chemical composition (ESI Figure S1). The average diameter of individual particles on the MFO and MZFO nanofibers was approximately 100 nm, with a single-crystal structure ranging from 90 to 105 nm (ESI Figures S2 and S3). MFO nanofibers were found to have a spinel structure, and Zn elements were successfully doped into the MFO nanofibers to form a single-phase MZFO with no impurity phase (ESI Figure S4). Figure 1a,b show the typical unit cell structure model of the ideal normal spinel MFO ferrite. It has a cubic unit cell structure containing 56 ions in total, in which the 32 O anions are closely packed, and 24 metal cations can be distributed in two different crystallographic sites, including a tetrahedral (A) site and an octahedral (B) site. As shown in Figure 1a, in the MFO unit cell ‘ball-and-stick’ model of the normal spinel structure, all the divalent Mg^2+^ cations occupy tetrahedral A sites, and all the trivalent Fe^3+^ cations occupy the octahedral B sites, in which the orange balls represent Mg cations (the atomic radius is 1.6 Å) and the bright blue spheres represent Fe cations (the atomic radius is 1.27 Å). Figure 1b shows the polyhedral model, which provides the corresponding polyhedral sites where different atoms are located. 

**Figure 2:**
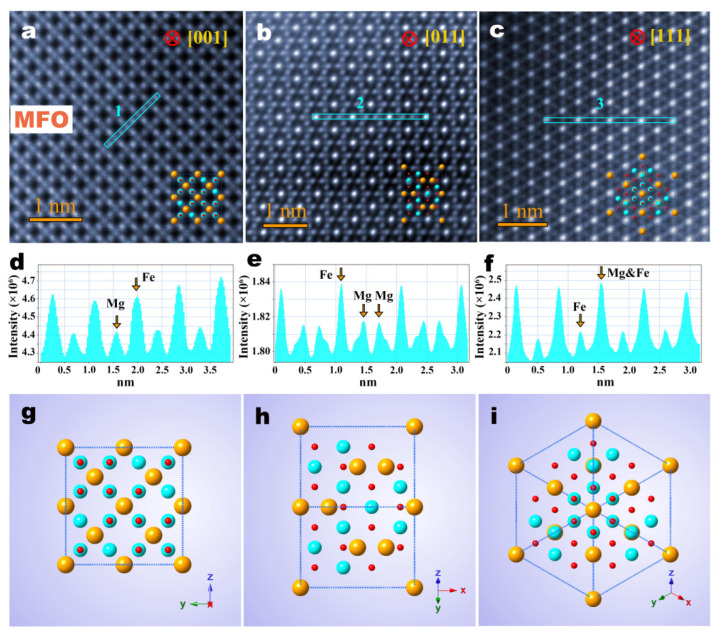
HAADF-STEM images and perspective views of normal spinel MFO structure. (**a**–**c**) The HAADF-STEM images of the MFO nanofibers projected along [001], [011] and [111] orientations, respectively. (**d**–**f**) Line intensity profiles for the atomic columns at tetrahedral A sites and octahedral B sites along blue line ‘1’, ‘2’ and ‘3’ in (**a**–**c**), respectively. (**g**–**i**) Perspective view of unit cells along [001], [011] and [111] orientations, respectively.

The atomic-scale cation occupations and distributions of individual MFO single-particle-chain nanofibers were then captured using Cs-STEM. As shown in Figure 2a–c, atomic-scale images of an individual MFO nanoparticle, along with [001], [011] and [111] orientations, were taken by HAADF-STEM (Cubed Titan G2 60-300, FEI, Hillsboro, OR, USA). 

It is noted that the contrast of the HAADF-STEM image is sensitive to the effective atomic number Z (Z_Mg_ < Z_Mg–Fe_ < Z_Fe_) and the number of atoms (the length of the column). The atomic number of iron (Z = 26) was significantly higher than magnesium (Z = 12) when the number of atoms on the atomic column was the same or similar, so the atomic columns of pure Fe and mixed Mg-Fe were much brighter than the pure Mg atomic columns. However, when the number of atoms on the atomic column greatly differed, the contrast of images of different atomic columns was sensitive to the number of atoms rather than the atomic number. Figure 1a shows that the Mg atomic column located in the tetrahedral sites is clearly darker than the surrounding Fe atomic column, and the Mg atoms (1.6 Å) are easily distinguished because they are larger in size than the Fe atoms (1.27 Å). The atomic images in the three crystal axes are all consistent with the cation occupancy and distribution of the ideal normal spinel structure. The corresponding unit cell models are shown in Figure 2g–i, which clearly show that all tetrahedral sites are occupied by Mg cations, and all octahedral sites are occupied by Fe cations. Figure 2d–f further show the line intensity profiles of ‘1’, ‘2’ and ‘3’ marked in the light blue color, in which ‘Mg’ represents the pure Mg atomic column at the tetrahedral A sites, ‘Fe’ represents the pure Fe atomic column in the octahedral B sites, and ‘Mg & Fe’ represents mixed Mg-Fe columns. The intensity distribution of the atomic columns further indicates that the Fe atomic columns at the octahedral B sites are brighter than the pure Mg atomic columns at the tetrahedral A sites, which is consistent with the theoretical atomic occupation of the ideal normal spinel MFO structure.

**Figure 3:**
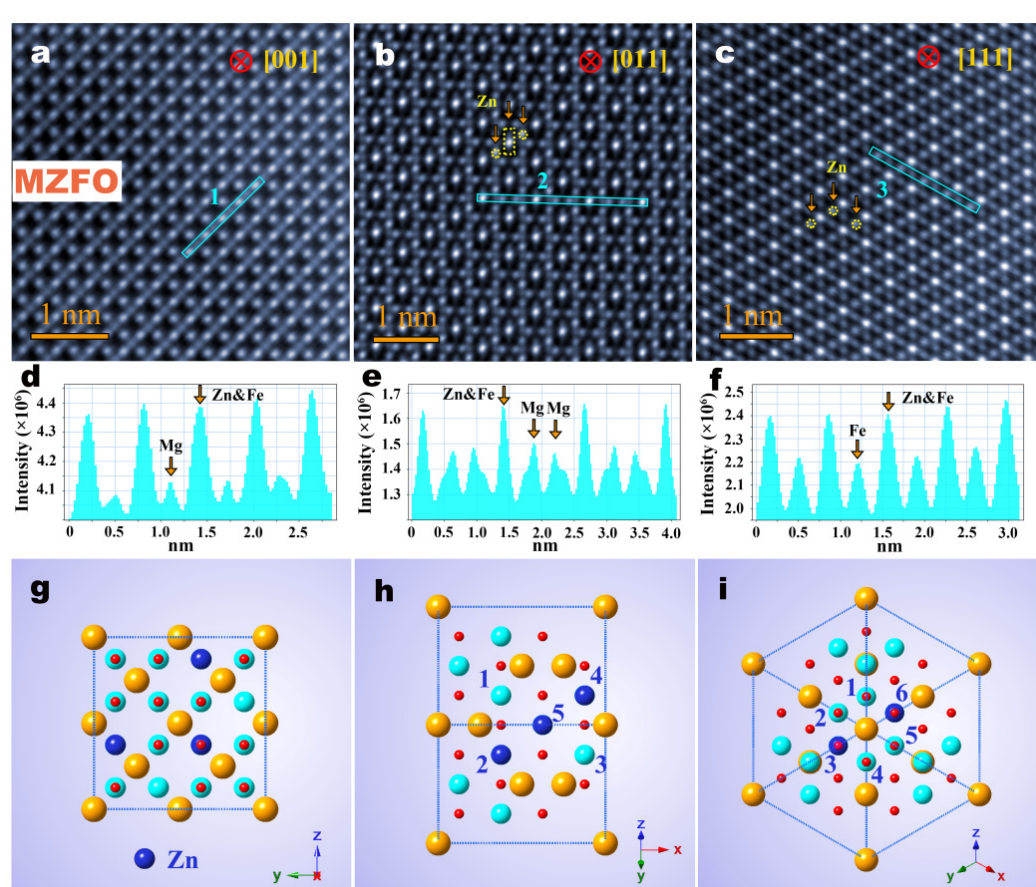
HAADF-STEM image and perspective views of Zn-doped normal spinel MFO. (**a**–**c**) Experimental HAADF-STEM images of MZFO nanofibers observed along the [001], [011] and [111] crystal ribbon axis directions. (**d**–**f**) The linear intensity distribution of the atomic column at tetrahedral A sites and octahedral B sites along the blue lines ‘1’, ‘2’ and ‘3’ in (**a**–**c**), respectively. (**g**–**i**) Perspective view of unit cells along [001], [011] and [111] orientations, respectively. The blue balls represent the Zn-doped atoms.

In parallel with the observed approach, the specific preferred sites of the dopant Zn atoms in the MFO nanofibers were further imaged. Figure 3a–c show typical HADDF-STEM atomic images of the MZFO nanofibers along the [001], [011] and [111] orientations. It is clear that MZFO nanofibers retain a spinel crystal structure in contrast to the pure MFO nanofibers. Figure 3a shows the atomic STEM image of the MZFO nanofibers projected along the [001] orientation, revealing that the overall atomic image contrast of MZFO nanofibers is essentially the same as that of the MFO nanofibers. It should be noted that the number of atoms on each column is the same in this direction, which presents an ideal situation to identify the preferred sites of dopant atoms. It can be seen that the brightness of the tetrahedral Mg atomic columns is nonetheless darker, but the octahedral Fe atomic column is brighter than that of the MFO. The line intensity distribution was obtained from the blue rectangular box ‘1’. As Figure 3d shows, the difference between the intensities of the two peaks is obviously larger than that of the MFO nanofibers (Figure 2d). Due to the atomic number Z (30) of the Zn atom being greater than the atomic number Z (26) of the Fe atom, the brightness of the octahedral atomic column increases. If the Zn atoms replace the Mg atoms at the tetrahedral sites, the brightness of the atomic column at the tetrahedral sites inevitably increases, thereby reducing the intensity difference between the two peaks. Furthermore, the atomic radius of a Zn ion (1.39 Å) and that of an Fe ion (1.27 Å) are relatively similar, making it easier for atomic substitution to occur. Therefore, it is believed that the dopant Zn atoms randomly replace the Fe atoms at the octahedral sites. The corresponding atomic unit cell model is shown in Figure 3g, in which the blue atoms represent the doping Zn atoms. Figure 3b shows the atomic image along the [011] crystal orientation, showing that the octahedral sites of the atomic column, marked by the yellow dashed circle in the image, are brighter than other octahedral atoms, and its corresponding sites are marked with the numbers ‘2’ and ‘4’ in the atomic unit cell model (Figure 3h). Moreover, the atomic tailing found around the Fe atoms at the octahedral sites, indicated by the yellow dashed rectangular box, may be caused by the irregular occupancy of the doping Zn atoms, which prevented the Zn-Fe atomic columns from being arranged in a straight line. The corresponding positions are indicated by the number ‘5’ in the atomic unit cell model (Figure 3h). Figure 3e is the line intensity distribution diagram shown in the blue rectangle ‘2’ in Figure 3b, and it indicates the specific distribution of the atomic column intensity at the tetrahedral and octahedral sites. Tilting the nanofiber to the [111] crystal axis, shown in Figure 3c, clearly shows that there are six octahedral Fe atomic columns around the Mg-Fe mixed atomic column, forming a regular hexagon, represented by the numbers ‘1–6’ in the atomic unit cell model (Figure 3i). These atomic columns should show the same brightness because each atomic column contains the same number of atoms. However, the octahedral atomic column marked by the yellow dashed circle in Figure 3c is obviously brighter than the surrounding columns. Its atomic unit cell model is indicated by the numbers ‘3’ and ‘6’ in Figure 3i. In addition, the line intensity analysis of the Fe atoms at the octahedral site marked by rectangle ‘3’ in Figure 3c further indicates the specific substitution position of dopant Zn atoms, which match well with the peak intensity distribution shown in Figure 3f and the unit cell model numbered as ‘1’ and ‘6’ atomic columns in Figure 3i. The Fe atom columns with the same number of atoms have different contrasts due to the substitution of Zn atoms, which makes pure Fe atom columns become Zn-Fe mixed-atom columns, further contributing to the increase in the brightness of the atomic column. The above results further prove that the MZFO nanofibers retain a normal spinel crystal structure, and the Zn substitutions occur in the Fe atomic columns at the octahedral sites. 

**Figure 4:**
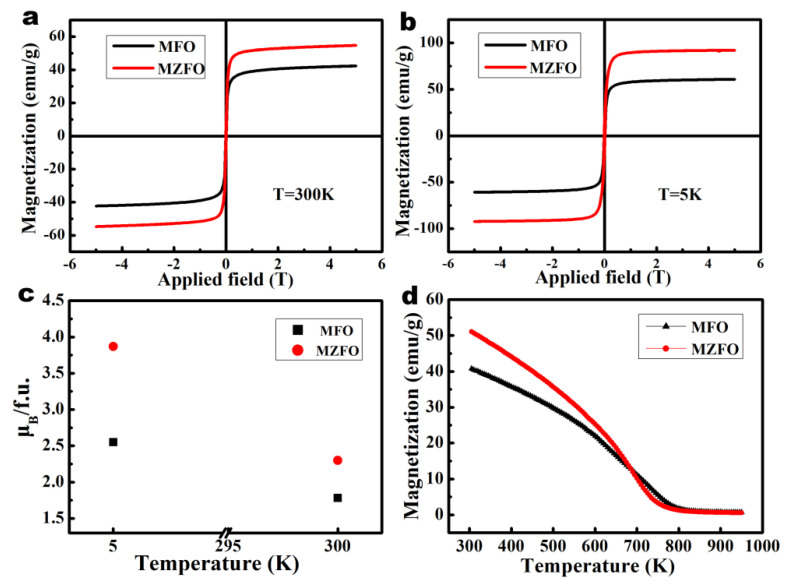
Magnetic properties of the spinel structure MFO and MZFO single-particle-chain nanofibers. (**a**) The hysteresis loops of MFO and MZFO single-particle-chain nanofibers measured under an external magnetic field of 3 T and a temperature of 300 K. (**b**) The hysteresis loops of MFO and MZFO single-particle-chain nanofibers measured under an external magnetic field of 3 T and a temperature of 5 K. (**c**) The plot of saturation magnetization (μ_B_/f.u.) vs. temperature of MFO and MZFO single-particle-chain nanofibers. (**d**) The saturation magnetization as a function of temperature measured at the 3 T external magnetic field and the temperature range of 300–950 K to obtain the Curie temperature.

In order to further verify the correlation between crystal structure and magnetic properties, SQUID and MPMS technologies were used to characterize the magnetic properties of the normal spinel MFO nanofibers and Zn-doped MFO nanofibers. The external magnetic field (3 T) dependence on the magnetization of the MZFO and MFO nanofibers at 300 K and 5 K is shown in Figure 4a,b, respectively. A quantitative analysis indicated that the saturation magnetization (*M*_s_) and coercivity (*H*_c_) of MZFO nanofibers at 300 K were 54.7 emu/g (2.30 μ_B_/fu) and 82 Oe, respectively, and the saturation magnetization (*M*_s_) and coercivity (*H*_c_) at 5 K were 92.2 emu/g (3.87 μ_B_/fu) and 145 Oe, respectively, compared to the pure MFO nanofibers with 42.3 emu/g (1.78 μ_B_/fu) 102 Oe at 300 K and 60.8 emu/g (2.55 μ_B_/fu) 167 Oe at 5 K. It was found that the saturation magnetization (μ_B_/f.u.) of MZFO nanofibers increased by 0.52 μ_B_/f.u. and 1.32 μ_B_/f.u. at 300 K and 5 K, respectively, and the coercivity had a slight tendency to increase.

The increase in saturation magnetization *M*_s_ and coercivity *H*_c_ should relate to the substitution of Zn^2+^ cations for Fe^3+^ cations at the octahedral sites, including the occupied sites; canted spin structure; and the super-exchange interaction between the cations and the O^2-^ anions [27], of which the exchange interactions should be the main cause. According to Neel’s theory of the sub-lattice [36], the cations located at the tetrahedral and octahedral sites indicate two sub-lattice positions, A and B, respectively, and the magnetic moments of cations at the tetrahedral A sites and the octahedral B sites are aligned in antiparallel in the spinel structure. The net magnetization (*M*_net_) is calculated by the following:

*M*_net_ = ∑*M*_B_ − ∑*M*_A_ (1)

where ∑*M*_B_ and ∑*M*_A_ represent the moment sum of cations located at octahedral sites and tetrahedral sites, respectively. The magnetization of the nanofibers depends on the distribution of the magnetic Fe^3+^ ions among the two sites because the Mg^2+^ and Zn^2+^ ions are non-magnetic.

The exchange interactions between two sub-lattices have different intensities; the antiferromagnetic A-B interaction is stronger than the ferromagnetic B-B interaction and A-A interaction, and the ferromagnetic A-A interaction is the weakest. In the normal spinel MgFe_2_O_4_ ferrites, all non-magnetic Mg^2+^ ions occupy the tetrahedral A sites, and all magnetic Fe^3+^ ions occupy the octahedral B sites. In the present study, Zn-doped MgFe_2_O_4_ ferrites, non-magnetic Zn^2+^ ions preferred occupying the octahedral Fe^3+^ site. A small amount of Zn^2+^ ions substituting Fe^3+^ ions increased the valence of the surrounding trivalent Fe^3+^ ions to Fe^3+x^ ions, which may have contributed to the increase in the saturation magnetization. The coercivity was related to magnetocrystalline anisotropy (*K*_1_ ~ −2.46 × 10^4^ erg/cm^3^ for MgFe_2_O_4_) and crystallite size in spinel magnetic ferrites. Therefore, the slight increase in coercivity could be attributed to the enhancement in magnetocrystalline anisotropy of ferrites due to the substitutions of Zn^2+^ ions in MgFe_2_O_4_ ferrite. The minor change in coercivity means that Zn doping does not change the soft magnetic properties of MgFe_2_O_4_ ferrite.

The Curie temperature of MFO and MZFO nanofibers was further measured in the temperature range of 300–950 K at the applied external magnetic field of 3 T, as shown in Figure 4d. The first order derivative was taken to find out the *T*_c_. It was evident that the *T*_c_ of MFO nanofibers was 725 K, which was higher than the value reported for MFO (713 K) in the literature [18]. When the Zn^2+^ ions were doped, the *T*_c_ of MZFO ferrite dropped to 690 K. The substitution of Zn ions for magnetic Fe ions reduced the Fe^3+^-O^2-^-Fe^3+^ super-exchange interaction, eventually lowering the Curie temperature. The paramagnetic Curie point *T*_p_ of the MFO and MZFO nanofibers were 904 K and 852 K, respectively, as shown in Figure 4d. When *T* > *T*_p_, the nanofibers were in a completely pure paramagnetic state.

## 4. Conclusions

In summary, we proved that the MFO nanofibers synthesized by electrospinning have a normal spinel structure by directly observing the occupancy and distribution of cations using Cs-STEM. The atomic HAADF-STEM images, combined with the theoretical atomic model and line intensity distribution, revealed that the dopant Zn^2+^ cations only randomly substitute for the Fe^3+^ sites rather than non-magnetic Mg^2+^ sub-lattices sites. Furthermore, the magnetic characterization showed that the Zn doping affects the spin states of the Fe^3+^, and the Fe^3+^-O^2−^-Fe^3+^ super-exchange interaction leads to an enhancement in magnetization and a reduction in the Curie temperature. We demonstrated that the doping of cations in spinel ferrites is an effective method to improve magnetic properties through tailoring the spin states of the cations and the occupation sites of the trace dopants, which provides guidance to modify the magnetic properties for the development of the next generation of multi-functional magneto-electric devices. 

## Data Availability

The datasets generated during and/or analyzed during the current study are available from the corresponding author on reasonable request.

## Supplementary Materials

The following are available online at https://www.mdpi.com/article/10.3390/ma15072422/s1.

## References

[1] Ferre R., Ounadjela K., George J.M., Piraux L., Dubois S. (1997). Magnetization processes in nickel and cobalt electrodeposited nanowires. Phys. Rev. B Condens. Matter.

[2] Satyanarayana L., Reddy K.M., Manorama S.V. (2003). Nanosized spinel NiFe_2_O_4_: A novel material for the detection of liquefied petroleum gas in air. Mater. Chem. Phys..

[3] Tartaj P., Morales M.D., Sabino V.V., Carreño T.G., Serna C.J. (2003). The preparation of magnetic nanoparticles for applications in biomedicine. J. Phys. D Appl. Phys..

[4] Peng J., Hojamberdiev M., Xu Y., Cao B., Wang J., Wu H. (2011). Hydrothermal synthesis and magnetic properties of gadolinium-doped CoFe_2_O_4_ nanoparticles. J. Magn. Magn. Mater..

[5] Rana S., Gallo A., Srivastava R.S., Misra R.D.K. (2007). On the suitability of nanocrystalline ferrites as a magnetic carrier for drug delivery: Functionalization, conjugation and drug release kinetics. Acta Biomater..

[6] Kotnala R.K., Shah J., Mathpal M.C., Gupta D., Purohit L.P., Kishan H. (2009). Role of modified active surface sites of magnesium ferrite for humidity sensing. J. Optoelect. Adv. Mater..

[7] Anantharamaiah P.N., Joy P.A. (2017). Tuning of the magnetostrictive properties of cobalt ferrite by forced distribution of substituted divalent metal ions at different crystallographic sites. J. Appl. Phys..

[8] Hemeda O.M., Mostafa N.Y., Abd Elkader O.H., Ahmed M.A. (2014). Solubility limits in Mn–Mg ferrites system under hydrothermal conditions. J. Magn. Magn. Mater..

[9] Patil J.Y., Mulla I.S., Suryavanshi S.S. (2013). Gas response properties of citrate gel synthesized nanocrystalline MgFe_2_O_4_: Effect of sintering temperature. Mater. Res. Bull..

[10] Kotnala R.K., Shah J., Gupta R. (2013). Colossal humidoresistance in ceria added magnesium ferrite thin film by pulsed laser deposition. Sens. Actuat. B.

[11] Khan M.A., Islam M.U., Ishaque M., Rahman I.Z. (2012). Magnetic and dielectric behavior of terbium substituted Mg_1−x_Tb_x_Fe_2_O_4_ ferrites. J. Alloys Compd..

[12] Iqbal M.J., Ahmada Z., Melikhov Y., Nlebedim I.C. (2012). Effect of Cu–Cr co-substitution on magnetic properties of nanocrystalline magnesium ferrite. J. Magn. Magn. Mater..

[13] Wang Y.C., Ding J., Yi J.B., Liu B.H., Yu T., Shen Z.X. (2004). High-coercivity Co-ferrite thin films on (100)-SiO_2_ substrate. Appl. Phys. Lett..

[14] Sivakumar N., Narayanasamy A., Shinoda K., Chinnasamy C.N., Jeyadevan B., Greneche J.M. (2007). Electrical and magnetic properties of chemically derived nanocrystalline cobalt ferrite. J. Appl. Phys..

[15] Reddy R.A., Rao K.R., Rajesh Babu B., Kumar G.K., Rajesh C., Chatterjee A., Jyothi N.K. (2019). Structural, electrical and magnetic properties of cobalt ferrite with Nd^3+^ doping. Rare Met..

[16] Xiang J., Shen X., Zhu Y. (2009). Effects of Ce^3+^ doping on the structure and magnetic properties of Mn-Zn ferrite fibers. Rare Met..

[17] Reyes-Rodrígues P.Y., Cortés-Hernándeza D.A., Escobedo-Bocardoa J.C., Almanza-Roblesa J.M., Sánchez-Fuentesa H.J., Jasso-Terána A., León-Pradoa L.E.D., Méndez-Nonellb J., Hurtado-López G.F. (2017). Structural and magnetic properties of MgZn ferrites (Mg_1−x_Zn_x_Fe_2_O_4_) prepared by sol-gel method. J. Magn. Magn. Mater..

[18] Ghodake U.R., Chaudhari N.D., Kambale R.C., Patil J.Y., Suryavanshi S.S. (2016). Effect of Mn^2+^ substitution on structural, magnetic, electric and dielectric properties of Mg-Zn ferrites. J. Magn. Magn. Mater..

[19] Fan W.Q., Li M., Bai H.Y., Xu D.B., Chen C., Li C.F., Ge Y.L., Shi W.D. (2016). Fabrication of MgFe_2_O_4_/MoS_2_ heterostructure nanowires for photoelectrochemical catalysis. Langmuir.

[20] West A.R. (1988). Basic Solid State Chemistry.

[21] Busca G., Finocchio E., Lorenzelli V., Trombetta M., Rossini S.A. (1996). IR study of alkene allylic activation on magnesium ferrite and alumina catalysts. J. Chem. Soc. Faraday Trans..

[22] Konishi K., Maehara T., Kamimori T., Aono H., Naohara T., Kikkawa H., Watanabe Y., Kawachi K. (2004). Heating ferrite powder with AC magnetic field for thermal coagulation therapy. J. Magn. Magn. Mater..

[23] Fatemeh S.M., Abdol M.D., Abbas R., Hamid B., Orhan Z., Barrett S.D. (2021). Structural, magnetic, and in vitro inhibitory characteristics of Ce-substituted MnFe_2_O_4_ nanoparticles. Appl. Phys. A.

[24] Davarpanah A.M., Rahdar A., Azizi Dastnae M., Zeybek O., Beyzaei H. (2019). (1−x)BaFe_12_O_19_/xCoFe_2_O_4_ hard/soft magnetic nanocomposites: Synthesis, physical characterization, and antibacterial activities study. J. Mol. Struct..

[25] Gao J.M., Cheng F.Q. (2018). Effect of preparation conditions on the structure and magnetic properties of metal-doped magnesium ferrites synthesized from laterite leaching solutions. J. Supercond. Nov. Magn..

[26] Rahman S., Samanta S., Errandonea D., Yan S., Yang K., Lu J.L., Wang L. (2017). Pressure-induced structural evaluation and insulator-metal transition in the mixed spinel ferrite. Phys. Rev. B.

[27] Dwivedi G.D., Tseng F., Chan L., Chatterjee S.L., Ghosh A.K., Yang H.D., Chatterjee S. (2010). Signature of ferroelectricity in magnetically ordered Mo-doped CoFe_2_O_4_. Phys. Rev. B.

[28] Manohar A., Krishnamoorthi C. (2017). Photocatalytic study and superparamagnetic nature of Zn-doped MgFe_2_O_4_ colloidal size nanocrystals prepared by solvothermal reflux method. J. Photochem. Photobiol. B Biol..

[29] Sawatzky G.A., Van Der Woude F., Morrish A.H. (1969). Mössbauer study of several ferrimagnetic syinels. Phys. Rev..

[30] Kedem D., Rothem T. (1967). Internal fields in nickel ferrite. Phys. Rev. lett..

[31] Wechsler B.A., Von Dreele R.B. (1989). Structure refinements of Mg_2_TiO_4_, MgTiO_3_ and MgTi_2_O_5_ by time-of-flight neutron powder diffraction. Acta Cryst..

[32] Moyer J.A., Vaz C.A.F., Negusse E., Arena D.A., Henrich V.E. (2011). Controlling the electronic structure of Co_1−x_Fe_2+x_O_4_ thin films through iron doping. Phys. Rev. B.

[33] Carta D., Casula M.F., Falqui A., Loche D., Mountjoy G., Sangregorio C., Corrias A. (2009). A structural and magnetic investigation of the inversion degree in ferrite nanocrystals MFe_2_O_4_ (M = Mn, Co, Ni). J. Phys. Chem. C.

[34] Subías G., Cuartero V., García J., Blasco J., Lafuerza S., Pascarelli S., Mathon O., Strohm C., Nagai K., Mito M. (2013). Investigation of pressure-induced magnetic transitions in Co_x_Fe_3−x_O_4_ spinels. Phys. Rev. B.

[35] Loukya B., Negi D.S., Dileep K., Pachauri N., Gupta A., Datta R. (2015). Effect of Bloch wave electron propagation and momentum-resolved signal detection on the quantitative and site-specific electron magnetic chiral dichroism of magnetic spinel oxide thin films. Phys. Rev. B.

[36] Neel L. (1948). Magnetic properties of ferrites: Ferrimagnetism and antiferromagnetism. Ann. Phys. Paris.

