# Supplementary material for: The Cation Distributions of Zn-doped Normal Spinel MgFe2O4 Ferrite and Its Magnetic Properties
Source: Materials (Basel). 2022 Mar 25;15(7):2422. doi: 10.3390/ma15072422 (PMC8999915; doi:10.3390/ma15072422)
Supplement: Supplementary file 1 [file materials-15-02422-s001.zip › materials-1613976-supplementary.pdf]

materials

The cation distributions of Zn-doped normal spinel $MgFe_2O_4$ ferrite and its magnetic properties

Xue Zeng, [1] Zhipeng Hou, [2] Jiaqi Ju, [1] Lei Gao, [3] Junwei Zhang [3,*] and Yong Peng [3,*]

[1] School of Mathematics and Physics, Lanzhou Jiaotong University, Lanzhou 730070, China; zengxue@lzjtu.edu.cn (X.Z.); 1194014153jjq@gmail.com (J.J.)
[2] Guangdong Provincial Key Laboratory of Optical Information Material and Technology & Institute for Advanced Materials, South China Academy of Advanced Optoelectronics, South China Normal University, Guangzhou 510006, China; houzp@m.scnu.edu.cn
[3] Electron Microscopy Centre of Lanzhou University and Key Laboratory of Magnetism and Magnetic Materials of the Ministry of Education, School of Materials and Energy, Lanzhou University, Lanzhou 730000, China; lgao19@lzu.edu.cn
* Correspondance: zhangjunwei@lzu.edu.cn (J.Z.); pengy@lzu.edu.cn (Y.P.)

Citation: Zeng, X.; Hou, Z.; Ju, J.; Gao, L.; Zhang, J.; Peng, Y. The Cation Distributions of Zn-doped Normal Spinel $MgFe_2O_4$ Ferrite and Its Magnetic Properties. *Materials* **2022**, *15*, x. https://doi.org/10.3390/xxxxx

Academic Editor : Dimitra Vernardou and Simone Pisana

Received: 11 February 2022
Accepted: 21 March 2022
Published:

Publisher's Note: MDPI stays neutral with regard to jurisdictional claims in published maps and institutional affiliations.

Copyright: © 2022 by the authors. Submitted for possible open access publication under the terms and conditions of the Creative Commons Attribution (CC BY) license (https://creativecommons.org/licenses/by/4.0/).

1. Analysis of the whole morphology of the MFO and MZFO single-particle-chain nanofibers.

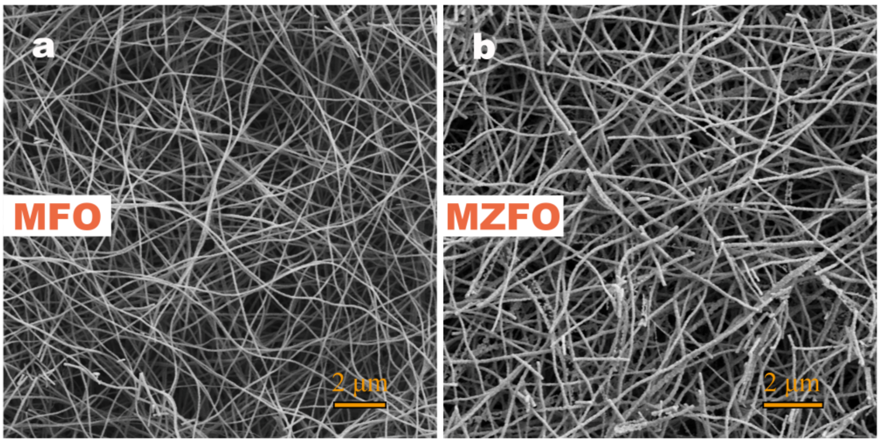

Figure S1. Morphological characterization of the MFO and MZFO single-particle-chain nanofibers. (**a–b**) Low-magnification SEM images showing the whole morphology of the MFO and MZFO nanofibers.

2. The morphology and chemical composition of the normal spinel structure of MFO single-particle-chain nanofibres.

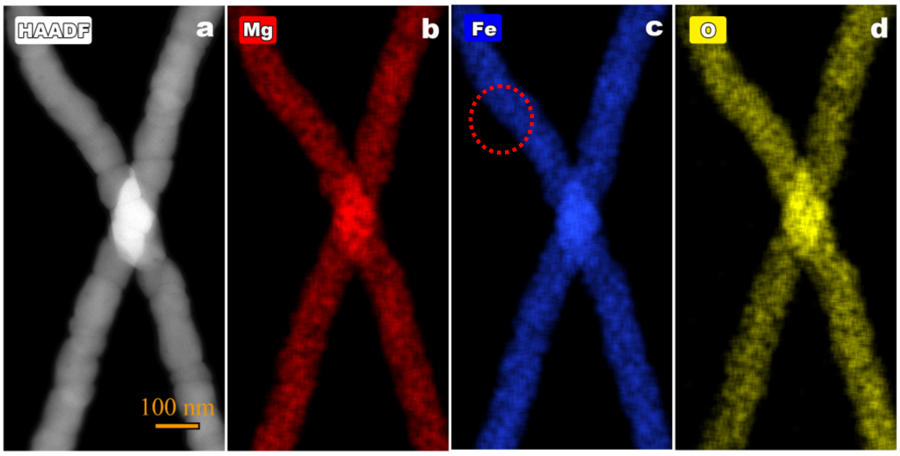

Figure S2. Morphological characterization of the normal spinel structure of MFO single-particle-chain nanofibers. (**a**) HAADF-STEM image of a single MFO single-particle-chain nanofiber. The particle circled in red was selected to take atomic images. (**b–d**) EDX elemental mappings of magnesium, iron and oxygen, respectively.

HADDF-STEM analysis proves that individual MFO nanofibre is composed of single particle stacked along the nanofibre axis in Fig. S2a. Fig. S2b–d show that representative elemental mappings of magnesium, iron and oxygen, respectively. The Mg, Fe and O elements are evenly distributed throughout the nanofiber, showing a uniform chemical phase.

3. The morphology and chemical composition of the normal spinel structure of MZFO single-particle-chain nanofibers.

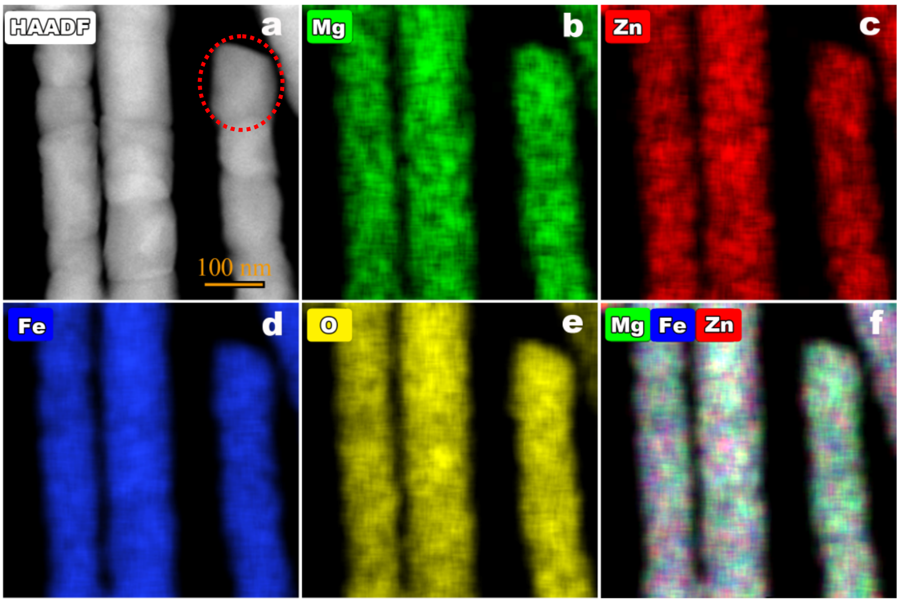

Figure S3. Morphological characterization of the normal spinel structure of MZFO single-particle-chain nanofibers. (**a**) HAADF-STEM image of a single MZFO single-particle-chain nanofiber. The particle circled in red was selected to take atomic images. (**b–e**) EDX elemental mappings of magnesium, zinc, iron and oxygen. (**f**) Mixed mapping image with Mg, Zn and Fe elements.

Fig. S3a shows a HADDF-STEM image of the MZFO single-particle-chain nanofibers. Fig. S3b–e display the corresponding EDX mappings of magnesium, zinc, iron and oxygen elements, respectively. It is seen that doping element Zn is uniformly distributed throughout the MFO nanofibers composed of a uniform chemical phase.

4. Chemical analysis of the normal spinel structure of MFO & MZFO nanofibers.

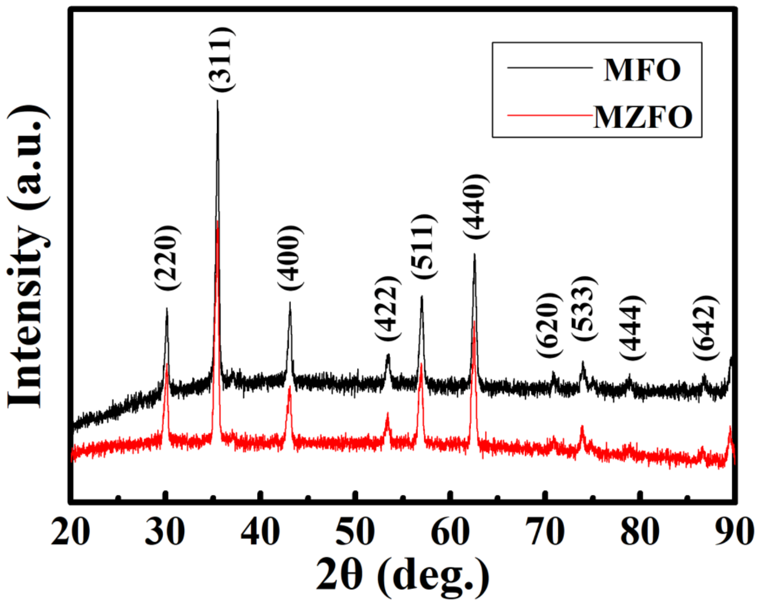

Figure S4. XRD spectrum of the $MgFe_2O_4$ and $Mg_{0.8}Zn_{0.2}Fe_2O_4$ nanofibers. Their peaks can be indexed to (220), (311), (400), (422), (511), (440), (620), (533), (444) and (642) planes, indicating a cubic spinel structure.

Fig. S4 shows two typical XRD spectra of a mass of the MFO and MZFO single-particle-chain nanofibers, which indicate that the Zn elements have been successfully doped into the MFO nanofibers, forming a single phase MZFO without no impurity phase.
